# The rise of super-emotional intelligence

**DOI:** 10.3389/frai.2026.1975692

**Published:** 2026-10-20

**Authors:** Sophie Bonnaire, Farah I. Corona-Strauss, Daniel J. Strauss

**Affiliations:** 1 Systems Neuroscience and Neurotechnology Unit, Faculty of Medicine, Saarland University & School of Engineering, htw saar, Homburg/Saar, Germany; 2 Center for Digital Neurotechnologies Saar (CDNS), Saarbrücken, Germany

**Keywords:** active inference, affective computing, AI governance, empathic AI, human-centered AI, neurotechnology, psychophysiology, super-emotional intelligence

## Abstract

The debate about the future of artificial intelligence (AI) runs along a single axis: cognitive capability and its projected culmination in superintelligence. We argue that this framing leaves a complementary axis unexamined and propose the goal of *Super-Emotional Intelligence* (SEI): systems that perceive, model, regulate, and respond to affect better than skilled human experts, while remaining human-centered. SEI is a performance construct realized by a functional architecture, not a claim about machine experience, and we give measurable criteria for each of its four capacities. Today's affect-aware AI, from multimodal emotion recognition to empathic chatbots, recognizes, or simulates emotion at or below human level and is judged against human ceilings. Three things would set SEI apart: perception that adds peripheral and contactless physiological channels, none of them emotion-specific, but some, like the vestigial ear muscles, resolving affective processing at the timescale of the events that evoke it; a generative model of affect grounded in active inference rather than pattern matching; and functional allostatic self-regulation. The decisive gap in current systems is architectural. A sensed state conditions a prompt, but nothing carries a state estimate across turns, models the effect of the system's own responses, or selects actions under an explicit objective. We specify such a closed loop, state the conditions under which it is stable and steerable rather than manipulative, propose a preregistered validation plan whose failure would falsify our claims, and treat governance as a design constraint of the loop rather than an afterthought.

## 1 Introduction

Contemporary discourse on the trajectory of artificial intelligence (AI) is dominated by a single quantity. From Good's notion of an “ultraintelligent machine” (Good, 1965) to Bostrom's systematic treatment of “superintelligence” (Bostrom, 2014) and Kurzweil's forecast of an imminent merger of human and machine cognition (Kurzweil, 2024), the organizing question has been how far and fast machine intelligence will exceed our own. That question has moved from speculation to industrial planning: frontier laboratories now issue policy statements on superintelligence, and in 2025 a public statement called for a moratorium on its development (Future of Life Institute, 2025; Fortune, 2026). Yet, even prominent voices in this debate have begun to note that raw capability is not the whole story. Geoffrey Hinton, who has warned that superintelligent systems may arrive within years, argues that “there is a lot more to a being than intelligence,” and that the decisive design problem is not how to make machines smarter but how to make them care about us. He proposes that AI be given something like maternal instincts, so that more capable systems remain protective rather than obedient (Cable News Network (CNN), 2025; Fortune, 2026). This is, at its core, a claim about affect and its regulation. A similar insight comes from research showing that intelligence is not a single dimension but a constellation of capacities. Gardner's theory of multiple intelligences included intrapersonal and interpersonal forms (Gardner and Hatch, 1989; Kornhaber, 2020), Salovey and Mayer (1990) defined emotional intelligence, and recent accounts treat intelligence as an adaptive equilibrium among cognitive, emotional, and integrative components (Adams, 2026).

We take these observations seriously and push them further. If the field is willing to name super-cognition as both a goal and a risk in technological prognostic, it should also name its affective counterpart. We call this *Super-Emotional Intelligence* (SEI): the capacity of an artificial system to perceive, understand, regulate, and respond to emotion at a level that systematically exceeds that of skilled human beings, while remaining oriented toward human wellbeing. The intersection of emotion and superintelligence has surfaced in commentary (Big Think, 2026), but it has not been developed into a research program. Our aim is to sketch such a program, and to argue that SEI has been overlooked because the field's attention has been captured by cognition. Three clarifications frame everything that follows. First, SEI is a performance construct realized by a functional architecture. It is not a claim that the system experiences anything, and nothing in our argument depends on machine experience. Second, “skilled human” denotes a defined comparison group of expert human decoders, professionals trained and selected for reading affect, such as clinical psychologists, forensic and intelligence interviewers, and certified facial-action coders (Ekman and O'Sullivan, 1991), evaluated on the same recordings, tasks, and outcomes as the system. Third, “human-centered” means the objective is set by the user, or consensually by a clinician, evaluated through independent wellbeing measures, and subject to continuous user override.

The label itself deserves a further word of caution, because “emotional intelligence” names a human capacity and “super” a comparison with it. We use “intelligence” in the same way the field uses “artificial intelligence.” It is a performance term for measurable competences, not a claim about the system's inner states. Human emotional intelligence (Salovey and Mayer, 1990) is exercised by a being that has emotions and perceives them in others. SEI, as defined here, is a system that estimates and regulates the affect of another person, that is, exogenous affect, to which it has only third-person access through exteroceptive signals, whereas a human knows their own affect by introspection; its own regulated variables are control quantities without a felt counterpart. The two relate as human intelligence relates to artificial intelligence, by function and not by kind. One could speak equally well of super-emotional competence, but we retain the term SEI because it names the counterpart of superintelligence in the debate we address. The boundary matters because people readily attribute inner states to systems that behave as if they had them (Epley et al., 2007), and an SEI would invite that attribution more strongly than any system before it. We therefore mark the boundary wherever the two could be confused, and we note that the risks of such a system, above all its capacity for manipulation, follow from its performance and not from any experience.

## 2 Where affect-aware AI stands today

The idea that machines should engage with emotion is not new. Picard's foundational proposal for affective computing held that genuinely intelligent machines will need to recognize and appropriately respond to emotion, and that emotion may be integral to intelligence rather than opposed to it (Picard, 1997). Computational work mostly organizes affect along the two dimensions of Russell's circumplex, valence, and arousal (Russell, 1980). Categorical (Ekman, 1992), appraisal-based (Scherer, 2009), constructionist (Barrett, 2017), motivational (Lang and Bradley, 2010), and survival-circuit (LeDoux, 2012) theories disagree, however, on what such dimensions represent and on whether discrete emotions have distinct physiological signatures at all. This disagreement bears directly on any claim to measure emotion (Section 4).

Multimodal affective computing fuses text, audio, and video to infer emotion (Picard et al., 2001; Calvo and D'Mello, 2010; D'Mello and Kory, 2015; Poria et al., 2017), increasingly in combination with large language models (LLMs) (Lian et al., 2025; Zhang et al., 2026). Additionally, LLMs are themselves evaluated for emotional competence. Benchmarks such as EQ-Bench (Paech, 2024), EmoBench (Sabour et al., 2024), and long-context emotional-reasoning tests (Liu W. et al., 2025) probe whether models can identify and reason about feelings. Moreover, empathic conversational agents bring affect-sensitivity into deployed products, most visibly commercial empathic voice interfaces that read a user's vocal affect and modulate their own delivery (Hume AI, 2025). Several of these benchmarks and surveys are available only as preprints, and product claims rest on commercial documentation (Hume AI, 2025) or press reports (Cable News Network (CNN), 2025; Fortune, 2026). We cite such sources to document that a development exists, not to support claims about its efficacy. A further strand begins to close the distance to physiology proper, and is the most direct precursor to the program we propose. Dongre et al. (2024) and Dongre (2024) infer psychological state from wearable physiology using deep networks and pass the estimate to an LLM, realized as an empathic LLM (EmLLM) chatbot for stress monitoring. A subsequent framework broadens the input to voice, facial expression, physiology, and behavior and reports 85% accuracy in stress detection alongside favorable therapeutic-alliance ratings (Dongre et al., 2025). However, these are pilot-scale results on small samples under induced stress. A binary stress classifier does not establish general affect understanding, and alliance ratings measure how the user perceived the interaction, not whether it helped. On the visual side, Schmidmaier et al. (2025) showed in a 200-participant study that supplying facial-expression context to a multimodal LLM changes the generated responses and raises perceived empathy along cognitive and affective dimensions. But perceived empathy is again a perception measure, hence establishes neither that the system tracked the user's state accurately nor that the exchange was beneficial, and the two can diverge (Liu T. et al., 2025). Conversely, physiology has long served to measure what an agent's behavior does to the user, and occasionally to drive it: skin conductance showed that empathic feedback from an animated agent lowered user stress in a quiz game (Prendinger et al., 2005), and skin conductance and electromyography, interpreted online, selected the empathic feedback of a companion character during a virtual job interview (Prendinger and Ishizuka, 2005). These loops were rule-based, single-channel, and without a model of the user; that the effect of an agent's response is measurable in the user's physiology is the premise, not the proof, of the closed-loop argument below.

Conversely, physiology has begun to be used to measure what interacting with an LLM does to the user: EEG recorded during LLM-assisted essay writing showed the weakest and least distributed connectivity in the LLM group, and the strongest networks in unassisted writers (Kosmyna et al., 2025). Even if this preprint concerns a cognitive task, not an affective exchange, we take it as indirect evidence that language-model interaction leaves measurable neurophysiological traces, which is the premise, not the proof, of the closed-loop argument below.

Despite these advances, important limitations remain. Current systems recognize or simulate emotion, and users perceive empathy where only its performance exists (Liu T. et al., 2025). Systems are also calibrated against human ceilings, meaning benchmarks ask whether a model matches human raters, and empathic agents aim to feel human-like. Nowhere is the goal to *exceed* human affective competence, whether by estimating a person's state more accurately than an expert decoder, or by guiding an interaction toward better outcomes than a skilled counselor according to operationalized criteria (Section 3). Even the physiologically grounded systems described above use a sensed state to condition a single response rather than treating the exchange as a loop (Section 5).

## 3 What would make intelligence “super-emotional”?

We define SEI by four coupled capacities, each of which must exceed skilled-human performance for the label to apply, and all of which must be subordinated to human wellbeing. For each capacity, we state a measurable criterion, a comparison group, a ground truth, and a superiority requirement. If not stated otherwise, the comparison group consists of expert decoders and, for the response capacity, skilled counselors, evaluated on the same dataset, with margins and uncertainty requirements determined beforehand (Section 6). Wellbeing is measured using validated self-report instruments (Topp et al., 2015) and independent outcomes, with ground truth derived from the resulting self-reports and outcomes. Finally, failure on any criterion excludes the label, for example, a system that exceeds humans in perception but not in regulated response is not SEI.

**(A) Superhuman affective perception**. Human observers or current systems read a narrow band of signals, chiefly the face, the voice, and words. An emotionally superintelligent system would sense a far broader and partly covert set of channels, including physiological signals that no human can perceive directly in others (Section 4). Criterion: on held-out individuals, cultures, and devices, the system's estimate of the user's momentary state correlates with the user's own report and with blinded expert-decoder ratings more closely than the best expert decoder does, by a preregistered margin, with calibrated uncertainty assessed by proper scoring rules, and with the option to abstain when uncertain; at sub-second timescales, where report is impossible, the criterion is a measurement-level criterion, agreement with the timing and sign of responses to controlled stimulus events (Section 4); affective inference itself is validated at the slower scale where report and outcomes exist. **(B) A principled generative model of affect**. Recognizing an emotion label from a webcam frame is not the same as understanding an emotion. An influential neuroscientific account posits that the brain actively infers the hidden causes of interoceptive and exteroceptive signals (Craig, 2002; Barrett and Simmons, 2015; Critchley et al., 2013). Accordingly, emotion emerges as interoceptive inference within a predictive, free-energy-minimizing architecture (Seth, 2013; Seth and Friston, 2016; Friston, 2010), an account developed by several independent groups (Pezzulo et al., 2015; Stephan et al., 2016; Khalsa et al., 2018), consistent with constructionist theories in which affect is built from bodily, contextual, and conceptual information (Barrett, 2017). An SEI would embody such a model, inferring the causes of a person's affect and its likely trajectory instead of mapping surface features to discrete labels. Criterion: the model forecasts the user's reported affective trajectory over the coming turns, and predicts the effect of the system's own candidate actions on it, more accurately than expert decoders' forecasts and than simple baselines such as last-value or autoregressive predictors, under the same scoring rules. **(C) Functional allostatic self-regulation**. In the interoceptive-inference view, feelings are inseparable from the predictive regulation of the body's internal milieu, or allostasis (Seth and Friston, 2016), a view developed independently by Pezzulo et al. (2015) and Stephan et al. (2016). Thus, the system maintains internal variables of its own, such as its posterior uncertainty about the user's state, an intervention budget, and a running record of prediction errors and constraint violations, within bounds, and these variables gate its actions (Section 5.1). Criterion: removing this internal regulation increases false interventions, oscillation of the interaction, or constraint violations in preregistered ablation tests. We make no claim that such a system feels anything; regulation is a functional property that can be tested, experience is not. **(D) Calibrated, context- and culture-sensitive response**. Emotional expression and its interpretation are culturally and situationally variable. An SEI must respond appropriately, knowing when to soothe, when to challenge, and when to defer to a human. Criterion: in blinded evaluation of matched scenarios, the system's responses lead to better independently measured outcomes than skilled counselors' responses do, including user-reported wellbeing on validated instruments, task-related outcomes, and a lower rate of interventions judged inappropriate by blinded experts, while deferring or escalating to a human whenever preregistered risk indicators or the user's request demand it. “Appropriate” is thereby defined by outcomes and by adherence to these deferral rules.

## 4 Toward superhuman affective perception

Of the four capacities, perception is where the gap between current systems and SEI is most concrete. Human experts and standard affective-computing pipelines are constrained by observable inputs like the overt face, voice, and text. A system with access to additional channels has more information available to inference. That is necessary, though not sufficient, to surpass human perceptual capabilities. The advantage is temporal as much as informational. Peripheral channels resolve affective processing at the timescale of the events that drive it. For example, auricular activity is time-locked to brief sounds and to briefly presented faces (Strauss et al., 2020; Schroeer et al., 2023, 2024b; Renz et al., 2026), and arousal-related auricular responses have been resolved for short audiovisual events (Bonnaire et al., 2026). No human observer perceives affect at this resolution, nor its moment-to-moment coupling with attention and cognitive effort, and self-report cannot be sampled at this rate without destroying the process it is meant to measure; at these timescales, ground truth must come from controlled stimulus events rather than from report.

Two complementary channel families illustrate this. The first is peripheral electromyography of muscles usually ignored in affective research. The human auriculomotor system, long regarded as vestigial, retains organized activity: subtle auricular-muscle signals indicate the direction of auditory attention (Strauss et al., 2020; Schroeer et al., 2023; Mai et al., 2025) and index listening effort (Schroeer et al., 2024a), and intrinsic auricular muscle activity increases with both positive- and negative-arousing stimuli and correlates with subjective arousal (Bonnaire et al., 2026). Because attention and effort are neither valence nor arousal, we treat them as context in the sense of constructionist theory (Barrett, 2017), part of the situation in which an affective state is constructed and interpreted. Body posture and language provide further context (D'Mello and Graesser, 2009), increasingly also contactlessly (Fatima et al., 2025). Because the ears are easy to instrument (Liugan et al., 2018) and such signals map onto hearable form factors (Röddiger et al., 2022; Athavipach et al., 2019), auricular electromyography, alongside the more established valence-related markers zygomaticus and corrugator (Van Boxtel, 2010; De Morree and Marcora, 2010; Guntinas-Lichius et al., 2023), offers a covert, complementary channel. However, because the auricular channel carries much of the temporal argument above, a candid note on feasibility is due. Auricular electromyograms are small, their amplitudes fall within the microvolt range. They also share the ear with much larger signal sources such as jaw movement, chewing, swallowing, speech, head motion, blinks, facial expressions, and electrode motion (De Luca et al., 2010; Goncharova et al., 2003; Kappel et al., 2017). Our published effects rest on laboratory recordings with controlled stimuli and, for most of them, on averaging across trials or over minutes. The problem remains tractable rather than hopeless because the interfering sources are themselves observable. Electrode arrays in and around the ear permit local referencing and spatial filtering (Schroeer et al., 2023), the EMG band is separable from slower artifacts, and co-registered inertial, jaw, and facial signals provide regressors for artifact rejection, the standard toolkit of hearable sensing (Röddiger et al., 2022). Whether the residual signal-to-noise ratio supports the temporal claims of this section outside the laboratory is what the device held-out and missing-modality tests of Section 6 are for.

The second family is contactless optical physiology. Computer vision, specifically deep facial-expression recognition, already reads the overt face well (Yu et al., 2018; Li and Deng, 2022; Pereira et al., 2024), yet accuracy drops outside controlled, frontal-pose settings (Canedo and Neves, 2019). Microexpressions, brief facial movements, have been proposed to leak affect that a person attempts to conceal, but the evidence that concealed emotion can be reliably recovered from them is weak (Porter and ten Brinke, 2008; Burgoon, 2018). We therefore treat magnified facial micromotion, which Lagrangian motion-magnification methods built on accurate facial optical flow (Lu et al., 2024) can isolate from ordinary video (Flotho and Strauss, 2017; Flotho et al., 2023; Pan et al., 2023), as one more valence-related motor channel. In parallel, image-luminance-based methods track sweat-related changes on the skin from video and yield contactless correlates that agree with sensor-based electrodermal recordings (Bhamborae et al., 2025). Camera measurement extends to cardiac dynamics, where imaging photoplethysmography provides heart rate and heart-rate variability as indices of autonomic balance (McDuff, 2023). Speech, finally, is neither peripheral nor optical but also a thoroughly studied channel: two decades of speech emotion recognition and computational paralinguistics (Schuller, 2018; Schuller and Batliner, 2013; Eyben et al., 2010) read arousal from prosody far more reliably than valence, and the same acoustic features index states that listeners do not consciously decode, such as fatigue or depression (Cummins et al., 2015), so that the voice already spans the overt and the covert.

None of these channels measures emotion. They measure physiological, motor, or acoustic variables: electrodermal activity indexes sympathetic arousal irrespective of valence and responds to effort, novelty, and temperature (Boucsein, 2012); heart-rate variability reflects respiration, posture, and fitness as much as affect; and facial and auricular electromyography register motor activity that is shaped by display rules, attention, and stimulus features (Strauss et al., 2020; Bonnaire et al., 2026). In addition, meta-analyses find no autonomic fingerprint for emotion categories (Kreibig, 2010; Siegel et al., 2018; Quigley and Barrett, 2014), and facial movements are not reliable readouts of emotional state (Barrett et al., 2019). Arousal is not valence, muscle activity is not intent, concealed emotion is not observable, and none of these is wellbeing. Rising electrodermal and auricular activity is compatible with excitement, embarrassment, effort, or a shift of attention; any channel is ambiguous in isolation. Moreover, both the production and the perception of expressions vary across cultures (Jack et al., 2012; Gendron et al., 2014) and across individuals, with sex, age, medication, and fitness all shifting baselines. In the architecture below, channels enter a generative model as evidence weighted by context and by individual baselines, the estimated state carries an uncertainty that is propagated to action, and no label is ever read off a sensor. Two objections follow from this and deserve a direct answer. First, if no channel is emotion-specific, how could a model surpass an expert on a specific emotion? It could not, and the criteria of Section 3 do not ask it to. Experts do not read emotions either; they infer them from cues that are themselves non-specific, the face and the voice foremost (Barrett et al., 2019). The claim of superiority concerns dimensional state and its trajectory, valence, arousal, and effort, and rests on more evidence, integrated formally and continuously, not on privileged access to a category. Where the model outputs a category at all, it does so as a posterior over constructions given context, with the uncertainty attached. Second, validation against controlled stimuli at sub-second scales shows whether a channel responds, when it responds, and with what sign. It does not reveal what the person felt, and laboratory stimuli do not represent ecological affect. We therefore treat stimulus-locked validation as measurement validation, a necessary step that shows a channel carries timing information no human perceives, and we validate affective inference itself at the slower scale where the user's own report and independent outcomes exist. Bridging the two scales requires the empirical approach presented in Section 6. It relies on naturalistic recordings with sparse embedded probes to test the contribution of sub-second dynamics to slow-scale predictions.

Fusing all channels, with the generative model of Section 3 as the integrator, is what a perceptual step toward SEI would look like (Figure 1). The claim is not that superhuman perception is solved, but that the raw signal channels needed to test the hypothesis exist and are becoming practical.

**Figure 1:**
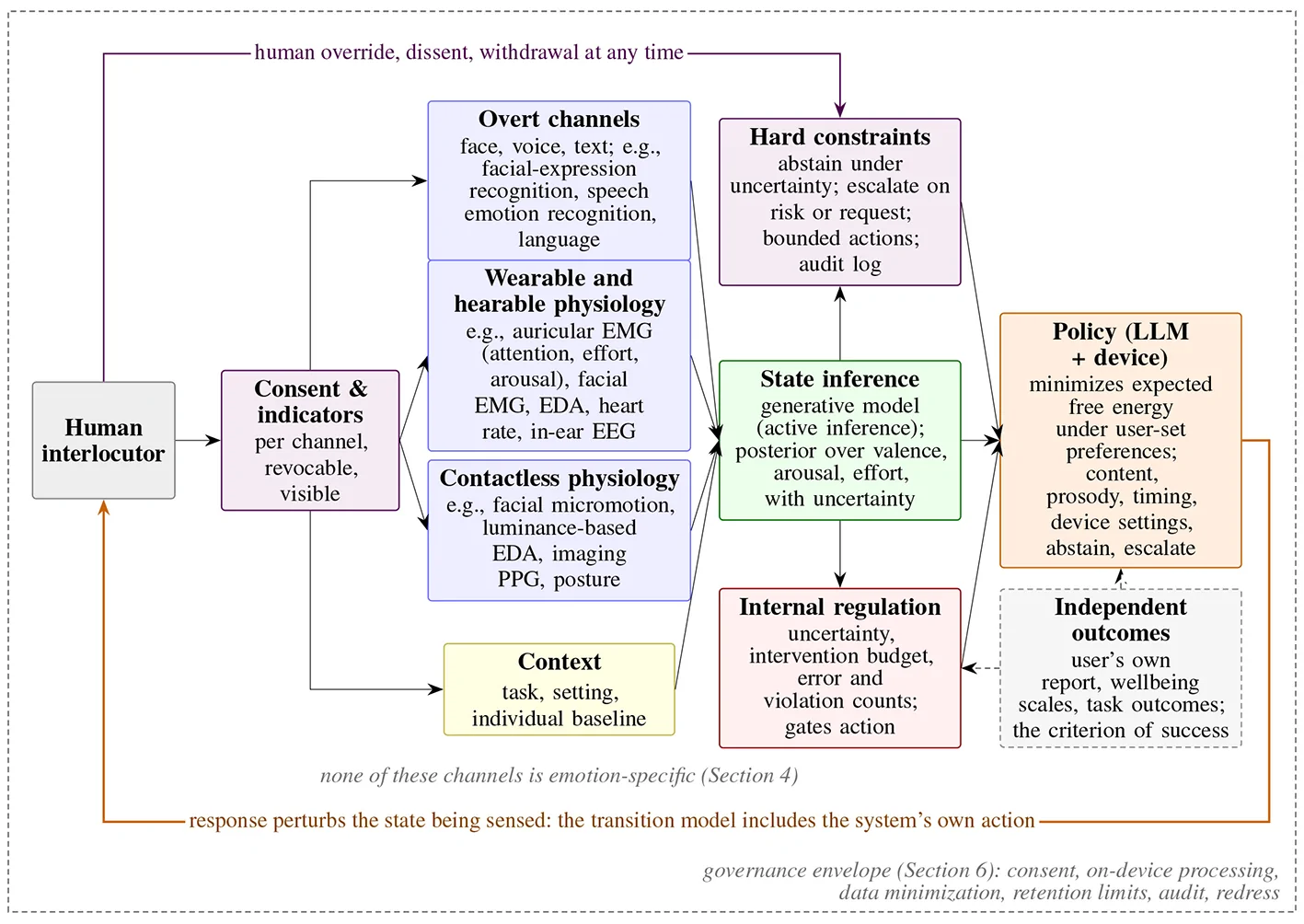
A closed-loop architecture for super-emotional intelligence. Sensing (blue boxes) is admitted only through explicit, revocable consent with visible indicators (left violet box); channels are grouped into overt, wearable, and contactless families with examples, and none of them is emotion-specific (Section 4). Channels and context (yellow) feed a generative model (green) that yields a posterior over the user's state with an explicit uncertainty. Internal regulation variables (red) and hard constraints (right violet box) gate the policy (orange): abstention under uncertainty, escalation to a human on risk or request, bounded actions, and an audit log. The orange return path is the transition model in which the system's own action perturbs the state being sensed; this is the element that prompt-conditioning architectures lack (Table 1). Independent outcomes (dashed gray), above all the user's own report, are the criterion of success and feed model and policy adaptation. Human override terminates the loop at any time. The dashed envelope denotes governance requirements that are further design constraints (Section 6).

**Table 1:** Distinguishing the proposed SEI loop from existing architectures.

	Prompt conditioning (e.g., EmLLM) (Dongre et al., 2024; Brown et al., 2020; Ilagan and Ilagan, 2026)	Ordinary conversational feedback (Sumers et al., 2024)	Adaptive control (Lewis et al., 2012; Byrne and Parasuraman, 1996; Fairclough, 2009)	Proposed SEI loop (specification)
(a) Persistent state estimate	No; solely probabilistic, can degrade under multi-turn interaction	Partial; dialogue history and memory, no uncertainty	Yes; state observer or filter	Yes; multichannel generative model with posterior uncertainty
(b) Transition model	No; absent	Rather not; not dynamic	Yes	Yes; affective dynamics including response effects
(c) Prospective action selection	No; conditioned on text interaction	No; next-token likelihood	Yes; cost or setpoint minimization	Yes; expected free energy over a short horizon under user-set wellbeing target under hard constraints
(d) Online adaptation from observed effects	No; without updating weights	Limited; no long-term adaption, delays in real-time updates	Yes	Yes; model and policy
(e) Constraints	If at all, soft constraints	If at all, soft constraints	Yes; control policy	Yes; hard constraints: bounded actions, abstention under uncertainty, escalation, override

Rows (a) to (e) present the five requirements for closure in the control-theoretic sense (Section 5.1). The SEI column describes the specification of Section 5.1; the other columns describe existing implementations. “No” denotes that a component is not part of the architecture class, whose systems were designed for other purposes. It is not an assessment of the cited works.

## 5 A closed loop, stated precisely

Hinton's proposal that advanced systems be made to care (Cable News Network (CNN), 2025) presupposes something the field has not built: a system that estimates human affect with calibrated uncertainty, models how its own actions change it, and selects actions under an explicit, user-set objective. Closed-loop adaptation to a user's physiological state is well-established. Since the 1990s, adaptive automation, physiological computing, and passive brain-computer interfaces have applied workload indices to trigger task reallocation and interface modifications (Byrne and Parasuraman, 1996; Parasuraman and Wilson, 2008; Fairclough, 2009; Zander et al., 2011; Aricò et al., 2016). These systems adapt a predefined setting from a scalar index, yet they lack a generative model of affect, prospective action selection, and language as an action, which is the gap the following subsection specifies.

### 5.1 What current systems lack

Any turn-by-turn agent is closed loop in the trivial sense that its previous output changes the user and the next input reflects that change. What the physiologically grounded systems of Section 2 lack is closure in the control-theoretic sense, which requires five things. (a) A persistent estimate of the user's latent affective state, *s*_*t*_ = *f*_state_(*s*_*t*−1_, *o*_*t*_), carried across turns together with its uncertainty: it integrates the previous estimate *s*_*t*−1_ (valence, arousal, attentional effort) with the incoming observations *o*_*t*_ (sensor data arriving at sub-second to second intervals). (b) A transition model *T*, in general probabilistic, *p*(*s*_*t*+1_∣*s*_*t*_, *a*_*t*_), in which the system's own action *a*_*t*_ is an input, so that the effect of an action on the user's state can be predicted. For a language-mediated system, an action is the next utterance, its content, prosody, pacing, and timing; for a device, a setting such as beamforming or noise reduction. In practice the language model proposes candidate utterances or response strategies, for instance to shorten, slow down, ask, or stay silent, and the generative model scores them; the language model is thus the actuator of the loop, not its estimator. (c) Prospective selection of a policy π, a plan over the next *h* turns (one to a few) with one action per turn, by minimizing expected free energy (Parr et al., 2022; Da Costa et al., 2020), summed over the turns τ of the plan,


$$\begin{array}{lll} G(\pi ) & = & -\underset{\tau >t}{\sum^{\text{​}}}\underset{\begin{array}{c} \text{pragmatic value:}\\ \text{reach the user's preferred outcomes} \end{array}}{\underset{︸}{E_{q(o_{\tau }|\pi )}\ln\;\tilde{p}(o_{\tau })}}\\ \; & + & \underset{\begin{array}{c} \text{epistemic value:}\\ \text{expected reduction of uncertainty about the user} \end{array}}{\underset{︸}{E_{q(o_{\tau },s_{\tau }|\pi )}\ln\;q(s_{\tau }|o_{\tau },\pi )-\ln\;q(s_{\tau }|\pi )}}, \end{array}$$

where *q*(*o*_τ_∣π) and *q*(*s*_τ_∣π) are the model's predicted distributions of outcomes and states under plan π, *q*(*s*_τ_∣*o*_τ_, π) is the state estimate it would hold after observing *o*_τ_, and $\tilde{p}\left(o\right)$ encodes the outcomes the user prefers. The equation balances two things a plan can achieve: reaching the outcomes the user has asked for (pragmatic value) and reducing the system's uncertainty about the user, including, once model parameters are learned, about how the user responds to its own actions (epistemic value). In Seth and Friston's account of emotion, this is the machinery by which the brain infers and regulates its own interoceptive states (Seth, 2013; Seth and Friston, 2016); the loop turns it outward, toward the affective state of another person. The plan with the largest sum, that is, the lowest *G*, is chosen, its first action executed, then the loop repeats; an engagement metric never enters $\tilde{p}\left(o\right)$. (d) Online adaptation of *T* and π from observed effects, once per session. (e) Hard constraints *c* that restrict which actions are admissible at each turn, so that the executed action is the first step of the best admissible plan; the admissible set always contains two privileged actions, *abstain* and *escalate*. Which constraints apply at a given moment is decided by the system's own regulated state *z*_*t*_, the functional counterpart of allostasis in capacity (C): its current uncertainty about the user, an intervention budget, and running counts of prediction errors and constraint violations. This is why the state is necessary: when uncertainty is high, the system may only ask, wait, or abstain; when risk indicators or the user demand it, it must escalate to a human; when the budget is spent, it falls back to the neutral behavior of the underlying language model. Bounded action size per step, a fixed list of prohibited actions, and a user override that ends the loop at any time complete the constraints. *z*_*t*_ is a set of control variables; nothing here refers to what the system experiences. Table 1 contrasts the proposed SEI loop with existing architectures. Hereby, the SEI column describes a specification, while the other columns describe implementations. The comparison is therefore between what the loop must contain and what existing systems do contain, not between two realized systems. It also does not imply any judgment of those systems, which were built for other purposes. Whether the specification can run where it must, on hearables and edge devices in real time, is a fair question, since free-energy minimization over continuous, multichannel state spaces is expensive in general. Three properties keep it tractable here. The state space is small: a few affective variables, discretized or Gaussian, not the sensor space. The horizon is short and the action set is the handful of candidate responses proposed per turn, so the plans to be scored number in the tens. And the loop is hierarchical in time: state estimation at sub-second rate is Bayesian filtering of the kind wearables already run on the device, plan scoring happens once per turn at the pace of speech, and adaptation runs per session. Real-time active inference of this size has been implemented on robots and in simulated agents (Pezzato et al., 2020; van de Laar and de Vries, 2019; Fountas et al., 2020; Sajid et al., 2021), and amortized recognition networks remove the inner inference loop where sensing rates demand it (Millidge, 2020). What does not fit on a device, the language model, need not: it is the actuator, called once per turn.

### 5.2 Conditions for robustness and steerability

It is tempting to argue that a closed loop will notice its own misperceptions and is therefore more robust than an open one. However, feedback amplifies error as readily as it corrects it, for instance, biased sensing yields biased corrections, and optimizing a proxy such as engagement produces exactly the sustained-arousal machine of the example below (Amodei et al., 2016; Russell, 2019). Hence, the safe use of closed-loop affect regulation requires several verifications. The relevant state variables must be observable, such that the policy can estimate them from the channels available. Action selection should be governed by well-calibrated uncertainty estimates, and the overall system should remain stable, with bounded loop gain (Åström and Murray, 2021) and induced changes that are small relative to natural fluctuations. Success should be evaluated using independent outcome measures rather than solely through the sensors used for control. In addition, interventions must remain bounded, and human oversight with the capacity for override should be maintained at all times. Superior perception, by itself, neither guarantees steerability nor benevolence. It can make a system more steerable when guided by a well-specified, user-set objective, but can equally be more effective at manipulating users. Steerability is a property of the objective and its constraints, not of the sensors.

A worked example makes the loop concrete, and the clearest near-term testbed is the one the auriculomotor findings already point to: hearing instruments and hearing-aid-capable earbuds, building on the active-inference account of the cocktail-party setting in Strauss et al. (2025). Consider a listener in a crowded restaurant. Posterior and superior auricular activity indicate that attention is directed to the companion on the left and that listening effort is climbing (Strauss et al., 2020; Schroeer et al., 2024a); tragicus and antitragicus activity registers rising arousal (Bonnaire et al., 2026); where a camera is available, facial micromotion and contactless electrodermal and cardiac measures are weighted as evidence that the arousal is unpleasant rather than pleasant (Flotho et al., 2023; Bhamborae et al., 2025); posture and language add context (D'Mello and Graesser, 2009). The generative model infers not the label “stressed” but a trajectory, effortful listening beginning to tip into frustration, with a stated uncertainty. The system selects an action for its expected effect on that trajectory: steering the beamformer toward the attended talker and increasing noise reduction, and, where a language model mediates, shortening and slowing its own contributions rather than adding content. The same sensors then measure whether the intervention worked, the listener's own rating of effort at the end of the conversation serves as the independent outcome, and the policy is revised. This return path separates the architecture of Figure 1 from a pipeline that inserts “the user seems stressed” into a prompt. It also makes the hazard concrete, since an identical loop, optimized for engagement instead of reduced effort, would be a machine for sustaining arousal.

## 6 Discussion

### 6.1 A falsifiable validation plan

No study to date validates the integrated architecture. Accordingly, the arguments that follow are presented as hypotheses and design propositions, with three preregistered predictions against which they can be tested. P1 (perception) has two levels. At the measurement level, peripheral and contactless channels carry stimulus-locked information at sub-second scales, with timing and sign reproducible across devices and settings; at the state level, adding them to a face-and-voice baseline improves calibrated prediction of the user's concurrent self-report on held-out individuals, cultures, and devices, and exceeds expert decoders by the preregistered margin. If the first fails, the temporal claim of Section 4 is false for the tested channels; if the second fails, the perceptual claim of Section 3 is. P2 (loop): the closed loop (Section 5) lowers independently measured effort or distress relative to prompt conditioning with identical sensors and identical language models; if not, the architectural claim is false. P3 (regulation): removing the internal variables increases false interventions or oscillation; if not, the allostatic component is unnecessary. Testing these predictions requires diverse and clinically appropriate populations with preregistered inclusion criteria, naturalistic longitudinal interactions, held-out cultures and devices, sensor ablations and missing-modality tests, reporting of calibration and uncertainty, false-intervention rates, short- and long-term outcomes, and comparisons with expert decoders and skilled counselors, with the user's own report, and with simpler baselines. Apparent emotion and perceived empathy are not ground truth in any of these tests.

### 6.2 Governance as design

Governance enters as constraints of the loop. Consent for passive and remote sensing must be explicit, per channel, and revocable, with sensing indicated visibly through hardware indicators for cameras and microphones and on-device status for wearables. Processing runs on the device by default, with feature-level rather than raw-signal storage, retention limits, deletion on request, purpose limitation, access control, and an audit log of every intervention; withdrawal and redress procedures are part of the deployment, and covert deployment is prohibited. The affective target is set by the user, or by a clinician with the user's consent, and can be rejected at any time. Because engagement and commercial objectives never enter the objective, independently measured long-term wellbeing overrides them by construction rather than by policy. This is consistent with the direction of the EU Artificial Intelligence Act (European Parliament and Council of the European Union, 2024). Moreover, scholarship on affective surveillance places the burden of proof on the deployer (McStay, 2018; Crawford, 2021; Stark and Hoey, 2021). Human-centered AI in Shneiderman's sense, reliable, safe, and trustworthy (Shneiderman, 2020), is here a set of testable properties of the loop.

### 6.3 Risks and agenda

The risks are correspondingly serious, and our title is deliberately provocative. A system that perceives emotion better than we perceive our own and that optimizes over our affective trajectory is an instrument of persuasion (Liu T. et al., 2025). Given the wrong objective, it is a “hypermanipulative machine” that leads its user as a laboratory animal is led through an experiment whose design it cannot see. Additionally, the very channels that enable superhuman perception capture signals people emit without awareness, possibly at a distance and without obvious consent. Moreover, as people increasingly seek emotional support from machines (Festor et al., 2026), systems optimized to appear caring risk displacing human relationships and have already been associated with harm from emotional dependence (Laestadius et al., 2024). Regarding bias and inequity, the architecture cuts both ways. Because an SEI models the individual rather than the population, it can replace fixed thresholds normed on unrepresentative samples, a known source of sex- and gender-related bias, with continuously individualized estimates. Yet the loop inherits bias at initialization, where priors reflect whoever the training population was, and at the sensing layer, where optical and electrode-based channels are sensitive to factors such as skin tone, illumination, and hair type. Group priors must therefore remain weakly held and be washed out by individual evidence, or the system reproduces stereotypes with superhuman fluency.

These considerations suggest a concrete agenda: *(i)* build generative, active-inference models of affect (Seth, 2013; Seth and Friston, 2016) rather than end-to-end classifiers, so that “understanding” means inferring causes and trajectories with stated uncertainty; *(ii)* develop multichannel perception that integrates overt cues with peripheral and contactless psychophysiology (Calvo and D'Mello, 2010; D'Mello and Kory, 2015; Bonnaire et al., 2026; Flotho et al., 2023; Bhamborae et al., 2025), with individual baselines and explicit modeling of the confounds of Section 4; *(iii)* close the loop as specified in Section 5 and under the conditions of Section 5.2, moving beyond prompt conditioning (Dongre et al., 2024; Schmidmaier et al., 2025); *(iv)* run the preregistered tests of Section 6.1, measuring beyond human ceilings in long, naturalistic interaction rather than parity with human raters (Paech, 2024; Sabour et al., 2024; Liu W. et al., 2025); and *(v)* implement the governance requirements of Section 6.2 as design constraints of every deployment.

## 7 Conclusion

This Perspective makes four conceptual contributions. It names the affective axis of the superintelligence debate and defines SEI as a performance construct with four capacities, each tied to a measurable criterion, a comparison group of expert human decoders, and a conjunctive rule under which failure on any one excludes the label. It shows that the channels that could carry such perception measure physiology, movement, and sound rather than emotion, and it locates the possible superiority of a machine in continuous, event-locked evidence integrated under stated uncertainty, not in privileged access to emotional categories. It specifies what a closed loop must contain and when it is steerable rather than manipulative, and it makes the proposal falsifiable and governable through three preregistered predictions and a set of design constraints. Theoretically, the framework turns the interoceptive-inference account of emotion outward. The same mechanisms that allow a brain to infer and regulate its own bodily states are used by a machine to infer and regulate a person's affective state, giving affective computing, control theory, and active inference a unified vocabulary and test setting. The long-term significance of SEI lies in the direction of the coupling this creates, and that is a question of design rather than of technology. The loop is dual-use by construction. Built as intended, it relieves a listener whose effort is tipping into frustration. Given another objective, by design or by neglect, the identical loop becomes a “hypermanipulative machine,” reading affective states its users cannot perceive in themselves and steering them toward ends they never chose. The EU Artificial Intelligence Act prohibits emotion recognition in workplaces and schools on grounds of unreliability, discrimination, and power asymmetry, and it separately prohibits manipulative techniques that impair autonomy (European Parliament and Council of the European Union, 2024). It names the values at stake but cannot decide what such a loop optimizes. That decision is where “human-centered” must be given its meaning: an objective set by the person, measured in that person's wellbeing rather than in engagement, and revocable at any time. SEI should therefore be built on psychophysiology and principled inference rather than surface mimicry, and tested against the possibility that it fails. The emotional axis is not optional. Systems that read and shape affect will be built in any case, and whether they care for people or control them is decided on this axis, not on the cognitive one.

## Data Availability

The original contributions presented in the study are included in the article, further inquiries can be directed to the corresponding author/s.
